# Neuron-specific biomarkers predict hypo- and hyperalgesia in individuals with diabetic peripheral neuropathy

**DOI:** 10.1007/s00125-021-05557-6

**Published:** 2021-09-03

**Authors:** Jakob Morgenstern, Jan B. Groener, Johann M. E. Jende, Felix T. Kurz, Alexander Strom, Jens Göpfert, Zoltan Kender, Maxime Le Marois, Maik Brune, Rohini Kuner, Stephan Herzig, Michael Roden, Dan Ziegler, Martin Bendszus, Julia Szendroedi, Peter Nawroth, Stefan Kopf, Thomas Fleming

**Affiliations:** 1 Internal Medicine I and Clinical Chemistry, University Hospital of Heidelberg, Heidelberg, Germany; 2 German Center for Diabetes Research (DZD), Neuherberg, Germany; 3 Medicover München Neuroendokrinologie, Munich, Germany; 4 Department of Neuroradiology, University Hospital of Heidelberg, Heidelberg, Germany; 5 Institute for Clinical Diabetology, German Diabetes Center, Leibniz Center for Diabetes Research at Heinrich Heine University Düsseldorf, Düsseldorf, Germany; 6 NMI Natural and Medical Sciences Institute at the University of Tübingen, Reutlingen, Germany; 7 Department of Molecular Pharmacology, Institute of Pharmacology, Heidelberg University, Heidelberg, Germany; 8 Institute for Diabetes and Cancer at Helmholtz Zentrum Munich, Neuherberg, Germany; 9 Division of Endocrinology and Diabetology, Medical Faculty, Heinrich Heine University, Düsseldorf, Germany

**Keywords:** Biomarker, Myelin protein zero, Myelination, Neurofilament light chain, Peripheral neuropathy

## Abstract

**Aims/hypothesis:**

The individual risk of progression of diabetic peripheral neuropathy is difficult to predict for each individual. Mutations in proteins that are responsible for the process of myelination are known to cause neurodegeneration and display alteration in experimental models of diabetic neuropathy. In a prospective observational human pilot study, we investigated myelin-specific circulating mRNA targets, which have been identified in vitro, for their capacity in the diagnosis and prediction of diabetic neuropathy. The most promising candidate was tested against the recently established biomarker of neural damage, neurofilament light chain protein.

**Methods:**

Schwann cells were cultured under high-glucose conditions and mRNAs of various myelin-specific genes were screened intra- and extracellularly. Ninety-two participants with type 2 diabetes and 30 control participants were enrolled and evaluated for peripheral neuropathy using neuropathy deficit scores, neuropathy symptom scores and nerve conduction studies as well as quantitative sensory testing at baseline and after 12/24 months of a follow-up period. Magnetic resonance neurography of the sciatic nerve was performed in 37 individuals. Neurofilament light chain protein and four myelin-specific mRNA transcripts derived from in vitro screenings were measured in the serum of all participants. The results were tested for associations with specific neuropathic deficits, fractional anisotropy and the progression of neuropathic deficits at baseline and after 12 and 24 months.

**Results:**

In neuronal Schwann cells and human nerve sections, myelin protein zero was identified as the strongest candidate for a biomarker study. Circulating mRNA of myelin protein zero was decreased significantly in participants with diabetic neuropathy (*p* < 0.001), whereas neurofilament light chain protein showed increased levels in participants with diabetic neuropathy (*p* < 0.05). Both variables were linked to altered electrophysiology, fractional anisotropy and quantitative sensory testing. In a receiver-operating characteristic curve analysis myelin protein zero improved the diagnostic performance significantly in combination with a standard model (diabetes duration, age, BMI, HbA_1c_) from an AUC of 0.681 to 0.836 for the detection of diabetic peripheral neuropathy. A follow-up study revealed that increased neurofilament light chain was associated with the development of a hyperalgesic phenotype (*p* < 0.05), whereas decreased myelin protein zero predicted hypoalgesia (*p* < 0.001) and progressive loss of nerve function 24 months in advance (HR of 6.519).

**Conclusions/interpretation:**

This study introduces a dynamic and non-invasive assessment strategy for the underlying pathogenesis of diabetic peripheral neuropathy. The diagnosis of axonal degeneration, associated with hyperalgesia, and demyelination, linked to hypoalgesia, could benefit from the usage of neurofilament light chain protein and circulating mRNA of myelin protein zero as potential biomarkers.

**Graphical abstract:**

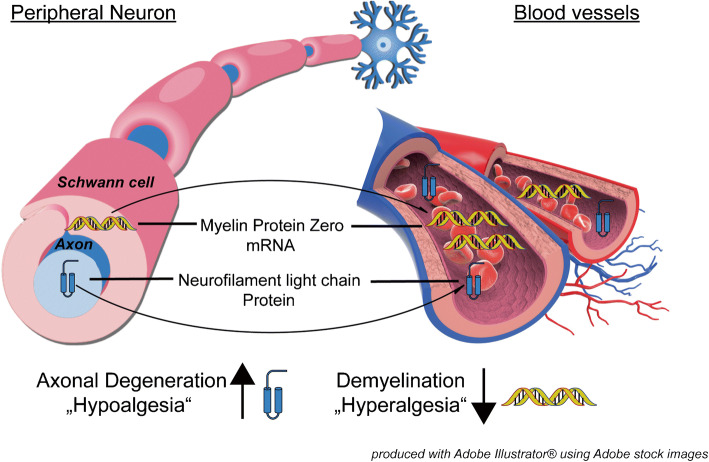

**Supplementary Information:**

The online version of this article (10.1007/s00125-021-05557-6) contains peer-reviewed but unedited supplementary material.

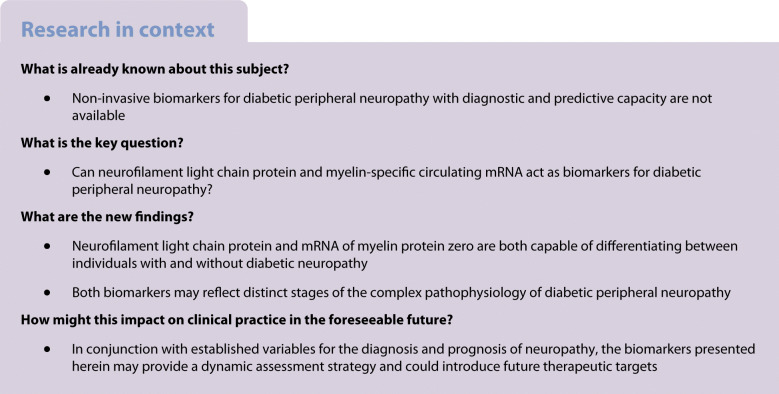



## Introduction

More than 50% of individuals with diabetes mellitus develop neuropathy, the major form of which is diabetic peripheral neuropathy (DPN). It is the most prominent risk factor for nontraumatic amputations, foot ulcerations and gait abnormalities, thereby resulting in an increased overall mortality risk of individuals with DPN [1]. Diagnosis of DPN is based primarily upon physical examination supplemented by symptom questionnaires, nerve conduction testing and/or quantitative sensory testing (QST) [2]. Up to two-thirds of patients have undiagnosed DPN, reflecting the urgent need for effective strategies for early detection of DPN by physicians [3]. However, when it comes to the structural integrity of nerve fibres, only indirect assumptions can be made. Clinical approaches such as neuronal conduction velocity (NCV) testing or magnetic resonance neurography (MRN) can help to differentiate between axonal and demyelinating damage in experimental settings, but are time consuming and costly [4, 5]. Furthermore, these diagnostic tests have very little predictive value on the progression of DPN, especially with regard to hypoalgesia, defined by the loss of sensorimotor function, which is a result of slow-developing NCV deficits in individuals suffering from DPN [6, 7].

From animal models it is known that DPN is to a high degree based upon demyelination of the nerve and dysfunction of Schwann cells. This can be the result of an altered expression of proteins responsible for the process of myelination, such as myelin basic protein (MBP), proteolipid protein 1 (PLP1), peripheral myelin protein 22 (PMP22) or myelin protein zero (MPZ) [8, 9]. Besides molecular-focused insights, studies on biomarkers in humans may add important knowledge in order to understand the physiology of nerve damage in diabetes [10, 11]. A novel biomarker-based approach derived from oncology is the use of circulating nucleic acids as a tool to screen and monitor patients at risk, of which its diagnostic and predictive capacities have been verified in several studies [12]. In diabetes, only a few studies have focused on circulating nucleic acids to discriminate disease severity [13, 14]. The assessment of nucleic acids that are solely expressed by cells of the peripheral nervous system could provide a degree of diagnostic specificity. This is based upon the assumption that genetic transcripts are protected inside the cell and, in the case of cell damage or other mechanisms (e.g. exosomal secretion), are released into the bloodstream by neuronal cells. Another promising biomarker in neurology is neurofilament light chain (NfL), a constituent of neurons and axons, which can be found in cerebrospinal fluid and, in trace amounts, in serum/plasma [15]. NfL offers an obvious benefit in monitoring the severity of neurodegenerative diseases, such as amyotrophic lateral sclerosis, Alzheimer’s disease and Huntington’s disease, but also of inherited neuropathies such as Charcot–Marie–Tooth disease, thereby reflecting the grade of the axonal damage [16].

In this prospective observational pilot study, we investigated the ability of myelin-specific circulating mRNA (cmRNA) and serum levels of an established neural damage marker, NfL protein, as potentially non-invasive tools for the detection of DPN and their capacity to predict the progression of the disease.

## Methods

### In vitro experiments

Human inducible pluripotent stem cells-derived Schwann (iPSC Schwann) cells were obtained from TempoBioscience (USA). Cells were grown in DMEM/F12 (Gibco, USA) with 17 mmol/l glucose and mannitol as osmotic control (66 mmol/l), 83 mmol/l glucose, 100 nmol/l insulin and 2.5% (v/v) NEFA supplement (Merck, Germany), containing also 10% FCS (Merck), 1% penicillin (10,000 units/ml) (Gibco), 1% streptomycin (10 mg/ml) (Gibco), 1% amphotericin B (250 μg/ml) (Gibco) and 1% glutamine, at 37°C in a saturated humidity atmosphere containing 95% air and 5% CO_2_. Culture dishes needed to be coated with poly-l-ornithine (10 μg/ml) and laminin (10 μg/ml) for 1 h prior to seeding of the cells. Cells were grown to 50–70% confluence for experiments and passaged at 90% confluence using 0.05% trypsin/EDTA (Gibco) for a maximum of five passages. Quantification of mRNA was achieved by RNA extraction (Peqlab, Germany, MicroSpin total RNA Kit) and conversion into cDNA (Thermo, USA, cDNA Reverse Transcription Kit), followed by quantitative PCR (qPCR) using ColorFlash SYBR Green Master Mix (Thermo) and a LightCycler480 (Roche, Switzerland). Relative expression levels were calculated using the ΔΔC_t_ method as described previously [17]. All primer sequences are described in Electronic supplementary material [ESM] Table 1. For western blot analysis and immunohistochemistry an antibody against MPZ was used (anti-rabbit, ab31851, Abcam, UK).

### Immunohistochemistry of human nerve material

Formalin-fixed paraffin-embedded human nerve sections of participants with and without DPN following lower limb amputation were obtained from the Department of Vascular Surgery, Heidelberg University Hospital and the Tissue Bank of the National Center for Tumor Diseases, Heidelberg as described previously [18]. Sections from the sciatic nerve (5 μm) were deparaffinated and antigen retrieval was performed using Target Retrieval Solution (DAKO, USA), according to the manufacturer’s instructions. Then, 0.5% saponin was used to permeabilise and 10% donkey serum in PBS-0.2% Triton X100 was used for blocking. Following an overnight incubation at 4°C with anti-MPZ antibody (1:100; Abcam), the sections were washed and incubated with anti-rabbit Alexafluor488 (1:1000; Cell Signaling, USA) for 1 h at room temperature. The sections were then washed and stained with DAPI (Thermo Fischer). Fluorescent images were taken on a confocal laser-scanning microscope (A1R; Nikon, Japan) and analysed using ImageJ software. Participant characteristics are provided in ESM Table 2.

### Study design

This single-centre, longitudinal, prospective observational study was performed from 4 February 2016 to 29 September 2018 at the Department of Endocrinology, Diabetes, Metabolic Diseases and Clinical Chemistry at Heidelberg University Hospital, Heidelberg. Study participants with and without type 2 diabetes were recruited from the diabetes outpatient care units of the Department. In total, 313 participants were screened, of whom 191 were excluded. Exclusion criteria were pregnancy; type 1 diabetes; end stage heart, liver or kidney failure; malignant diseases; chronic alcohol abuse; prolapsed discs; systemic or neurological diseases that are associated with neuropathy; ongoing medication possibly inducing neuropathy; and any other causes of neuropathy such as hypovitaminosis, monoclonal gammopathy, exposure to neurotoxic agents, Parkinson’s disease, restless legs syndrome or multiple sclerosis. Eligibility criteria included age 35–85 years and diagnosis of type 2 diabetes 3 years before enrolment or earlier. Participants with type 2 diabetes were enrolled if the diagnosis had been established according to the guidelines of the German Diabetes Association [19]. Glucose tolerant participants underwent an oral glucose tolerance test to confirm plasma glucose level below 7.8 mmol/l following ingestion of 75 g of glucose. The follow-up period comprised two visits, 12 and 24 months after enrolment. All participants were similar in age, BMI and gave written informed consent and the study was approved by the local ethics committee of Heidelberg University (No. S146-2015).

### Clinical chemistry

At baseline and after 12 and 24 months, blood was drawn and urine was taken under fasting conditions in the morning and samples were immediately processed in the accredited Central Laboratory of Heidelberg University Hospital. Plasma and/or urine levels of creatinine, cystatin C, total cholesterol, lipoprotein a, HDL-cholesterol, triacylglycerols and albumin in all participants were analysed with clinical chemistry automation (AdviaXPT 2400 chemistry analyser, Siemens Healthineers, Germany) according to the appropriate standard operating protocol. Albumin/creatinine ratio (urinary ACR) was calculated in mg/g and values >30 mg/g were defined as pathological. The Chronic Kidney Disease Epidemiology Collaboration (CKD-EPI) formula was used for the estimation of GFR [20].

### Assessment of DPN

All participants were examined using neuropathy deficit score (NDS) and neuropathy symptom score (NSS) as described previously [21]. For the QST, a protocol was established using seven different tests with 13 different categories for determination of ‘gain of function’ (hyperalgesia) or ‘sensory loss’ (neuropathic deficits) as described previously [21]. Electrophysiological examination was performed in 119 participants using a Viking IV electromyography system (Viasys Healthcare, France) on peroneal, tibial and sural nerves. All tests were performed on one foot to detect distal neuropathic deficit and on one hand as an intra-patient control area. All tests were performed at baseline and repeated after 12 and 24 months by investigators trained and certified by the Department of Neurophysiology at the University Hospital of Mannheim. DPN was determined by a score of 3 or higher in the NDS and NSS and abnormal electrophysiological examinations in two different nerves as described previously [21]. In addition, 37 participants, of whom ten were control participants and 27 participants with type 2 diabetes (21 with DPN), underwent diffusion-weighted high-resolution MRN of the right thigh in a 3.0 Tesla MRI scanner (Magnetom TIM-TRIO; Siemens Healthcare, Germany; 15-channel transmit–receive extremity coil) at baseline in order to determine the sciatic nerve’s fractional anisotropy (FA), which was calculated in an automated approach using the Food and Drug Administration (FDA)-approved software ‘Nordic BRAINEX’ (Version 2.2, https://nordicneurolab.com/nordicbrainex/, Nordic Neurolab, Bergen, Norway) [22, 23]. A detailed description for each of the methods used for assessing DPN is provided in the ESM Methods.

### cmRNA measurements

Serum blood samples were collected from all participants and centrifuged within 1 h. For the quantification of circulating myelin-specific mRNA, a previously established direct-serum qPCR technique was used with minor changes as described in various cancer studies [24–26]. Detailed descriptions of the extraction technique and calculation as well as primer sequences and primer pair efficiency for the analysis of cmRNA content can be found in the ESM (ESM Table 1, ESM Fig. 1).

### External validation study

A detailed description can be found in the ESM.

### Quantification of NfL protein

A detailed description can be found in the ESM Methods.

### Statistical analysis

Statistical data analysis was performed using GraphPad Prism 7 (https://www.graphpad.com/scientific-software/prism/, GraphPad Software, USA) and SPSS Version 23.0 (https://www.ibm.com/analytics/spss-statistics-software, IBM, USA). A detailed description can be found in the ESM Methods.

## Results

### Study population

**Fig. 1:**
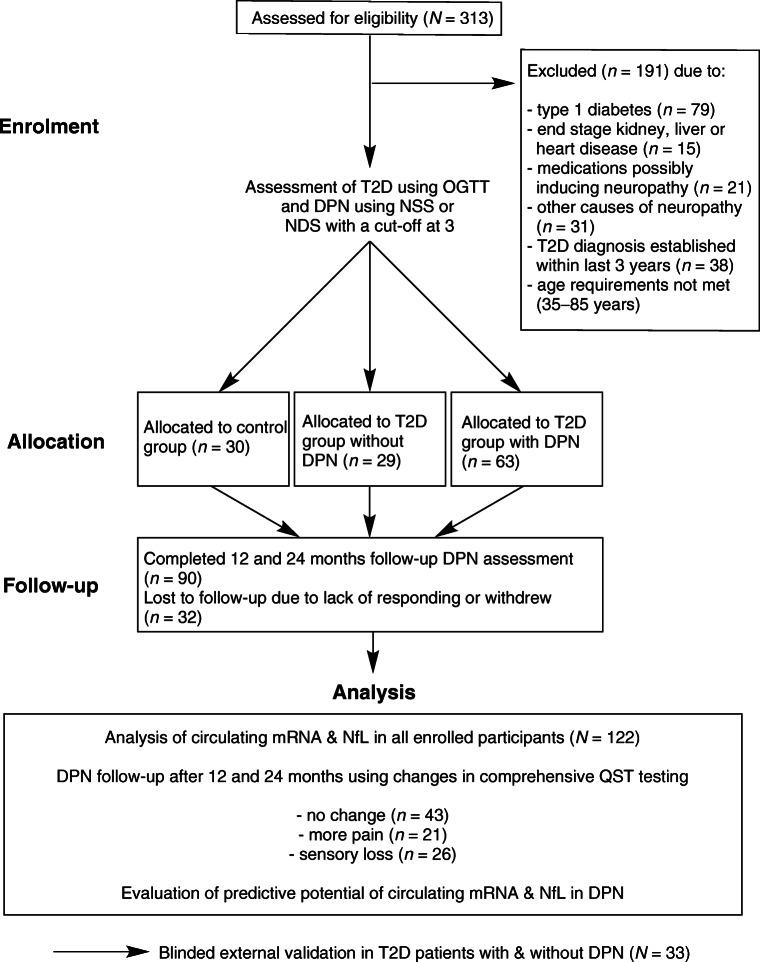
Flow of participants. T2D, type 2 diabetes

After enrolment the study cohort consisted of 122 participants, of whom 30 were classified as control participants, 29 as participants with type 2 diabetes without DPN and 63 as participants with type 2 diabetes with DPN (Table 1, Fig. 1). Between the groups, no significant differences were found regarding age, sex distribution, eGFR, lipoprotein a [Lp(a)], blood pressure, C-reactive protein and plasma cholesterol levels. Participants with type 2 diabetes showed higher fasting blood glucose, HbA_1c_ and triacylglycerols as compared with control participants (Table 1).

**Table 1:** Baseline demographic, laboratory and clinical profiles of study participants

Variable	Control group (*n* = 30)	w/o DPN (*n* = 29)	With DPN (*n* = 63)
Age	58.0 ± 13.38	61.9 ± 11.17	62.62 ± 9.18
Sex (f/m)	16/14	14/15	23/40
Diabetes duration (years)	–	11.4 ± 8.9	11.9 ± 8.1
BMI (kg/m^2^)	28.6 ± 5.7	30.9 ± 7.1	31.0 ± 5.8
Fasting glucose (mmol/l)	6.16 ± 0.81	8.10 ± 0.84^*^	8.31 ± 1.74^†^
HbA_1c_ (mmol/mol)	41 ± 3.3	53 ± 11.0^*^	54 ± 14.3^†^
HbA_1c_ (%)	5.9 ± 0.3	7.0 ± 1.0^*^	7.1 ± 1.3^†^
eGFR (ml min^−1^ 1.73 m^−2^)	95.8 ± 9.8	91.6 ± 18.6	83.6 ± 21.1
hsCRP (mg/l)	1.7 ± 2.9	3.1 ± 2.9	2.9 ± 3.0
Lp(a) (g/l)	0.286 ± 0.259	0.281 ± 0.276	0.253 ± 0.294
Triacylglycerols (mmol/l)	1.07 ± 0.42	1.71 ± 0.71^*^	1.99 ± 0.93^†^
Cholesterol (mmol/l)	5.39 ± 1.05	5.04 ± 0.99	4.69 ± 1.08
BP systolic (mmHg)	130.4 ± 18.5	139.2 ± 18.8	138.2 ± 15.9
BP diastolic (mmHg)	82.4 ± 11.2	84.0 ± 13.2	87.3 ± 9.8
NSS	–	–	5.4 ± 2.6^†‡^
NDS	–	–	5.9 ± 3.0^†‡^
Oral antidiabetics	0 (−)	15 (52)^*^	37 (59)^†^
Insulin therapy	0 (−)	6 (21)^*^	17 (27)^†^
RAAS inhibitors	1 (3)	12 (41)^*^	40 (63)^†^
Beta blockers	2 (7)	8 (28)^*^	21 (33)^†^
ASA	4 (13)	5 (17)^*^	18 (29)^†^
Statins	4 (13)	5 (17)^*^	25 (40)^†‡^
Diabetic nephropathy	–	6 (21)^*^	5 (8)^†^
Diabetic retinopathy	–	–	–

Data are mean ± SD or *n* (%)

–, data is not available

^*^p < 0.05 control participants vs participants without DPN

^†^*p* < 0.05 control participants vs participants with DPN

^‡^*p* < 0.05 without DPN vs with DPN

ASA, acetylsalicylic acid; f, female; hsCRP, high-sensitive C-reactive protein; m, male; RAAS, renin–angiotensin–aldosterone system; w/o, without

### Myelin-specific cmRNA

**Fig. 2:**
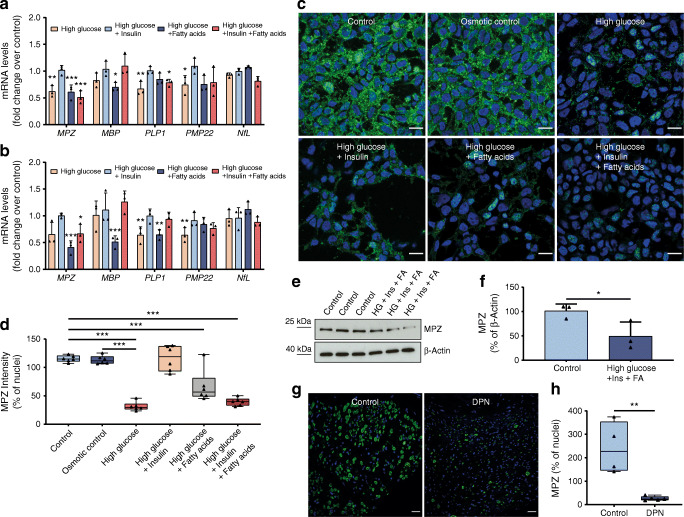
Expression of MPZ is affected in experimental and clinical diabetes. (**a**) Intracellular mRNA expression in iPSC Schwann cells treated with high glucose (83 mmol/l), high glucose + insulin (100 nmol/l), high glucose + fatty acids (2.5%) and high glucose + insulin + fatty acids for 72 h. (**b**) mRNA content in the culture medium of iPSC Schwann cells treated with four conditions for 72 h as described in (**a**). (**c**) Immunofluorescence staining for MPZ in iPSC Schwann cells treated with high glucose (83 mmol/l), mannitol (66 mmol/l), high glucose + insulin (100 nmol/l), high glucose + fatty acids (2.5%) and high glucose + insulin + fatty acids (2.5%) for 48 h (scale bar, 20 μm). (**d**) Analysis of fluorescence intensity of MPZ (green) relative to the nuclei (blue) in iPSC Schwann cells as shown in (**c**). (**e**) Western blot analysis of total cell extracts (30 μg of protein lysates) from iPSC Schwann cells treated with high glucose + insulin + fatty acids and probed with anti-MPZ after 48 h; expected band size for MPZ is 27 kDa. (**f**) Densitometry analysis of western blot as described in (**e**). (**g**) Representative immunofluorescence staining for MPZ in human sciatic nerve sections from control participants and participants suffering from DPN (scale bar, 100 μm). (**h**) Analysis of fluorescence intensity of MPZ (green) relative to the nuclei (blue) in human sciatic nerve sections from control participants (*n* = 4) and participants suffering from DPN (*n* = 5) as shown in (**g**). All mRNA data are normalised to β-actin and represent the mean fold-change over osmotic control (mannitol) ± SD; *p < 0.05; **p < 0.01; ***p < 0.001. Ins, insulin

**Fig. 3:**
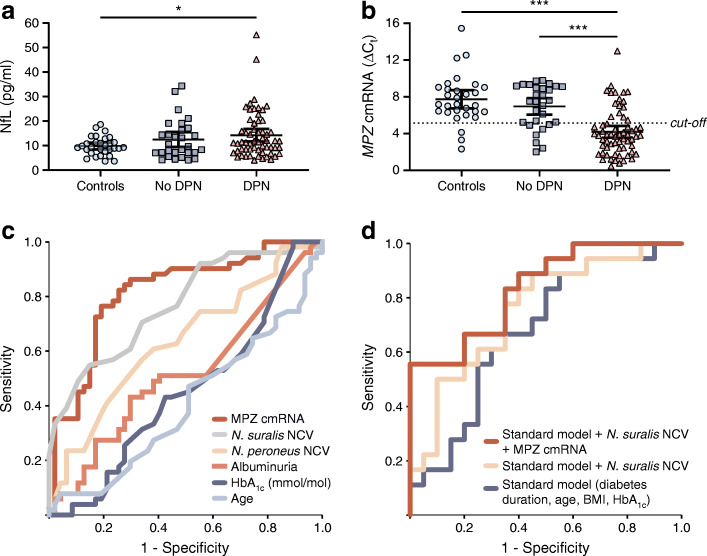
Diagnostic performance evaluation of NfL and cmRNA of *MPZ*. (**a**) Quantification of serum NfL levels in control participants (*n* = 30), participants with type 2 diabetes without DPN (*n* = 29) and participants with type 2 diabetes and with DPN (*n* = 60). (**b**) Quantification of serum *MPZ* cmRNA in control participants (*n* = 30), participants without DPN (*n* = 29) and participants with DPN (*n* = 63). The dashed line represents the optimal cut-off value as 5.08 to discriminate between participants with or without DPN. (**c**) Comparison of different ROC curve analyses for discriminating participants with DPN from participants without DPN using age, HbA_1c_, albuminuria, NCV of *N. peroneus* and *N. suralis*, and *MPZ* cmRNA. (**d**) Comparison of combined ROC curve analyses for discriminating participants with DPN from participants without DPN using a standard model (diabetes duration, age, BMI, HbA_1c_), a standard model with NCV of *N. suralis* and a standard model with NCV of *N. suralis* and *MPZ* cmRNA. Characteristics of ROC curves are summarised in Table 2. All *MPZ* cmRNA data represent the ΔC_t_ values normalised to the geometric mean of four individual reference genes (*UBC*, *eEF1a1*, *GAPDH*, *18S*). Serum NfL and *MPZ* cmRNA data are displayed as mean value with 95% CI; *p < 0.05; ***p < 0.001

**Fig. 4:**
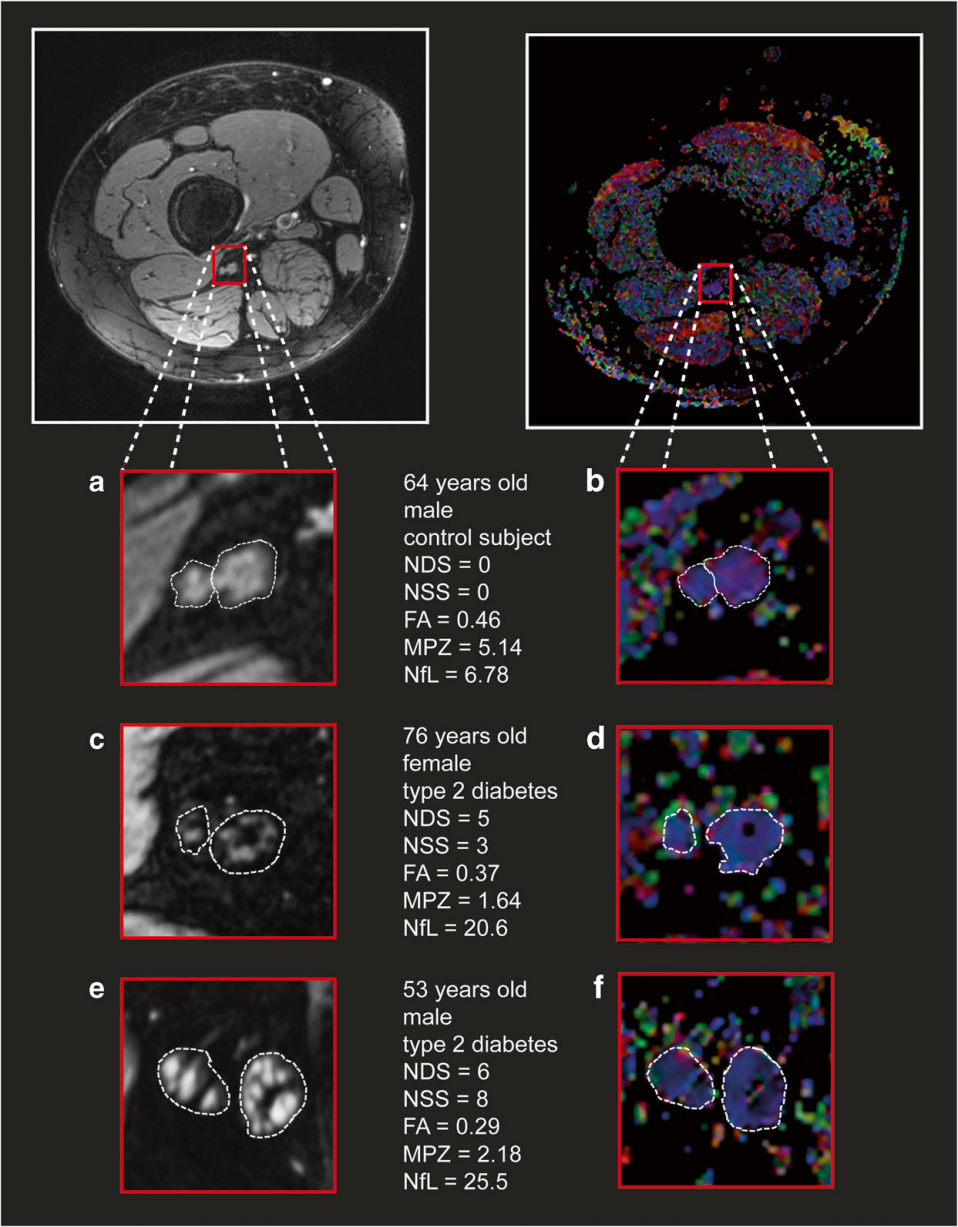
FA displays the integrity of nerve fibre mass in human sciatic nerve. (**a**) T2-weighted, fat-suppressed image of tibial and peroneal compartments of the sciatic nerve in a healthy control participant. (**b**) Colour-coded map of the FA of tibial and peroneal compartments of the sciatic nerve of the same participant as in (**a**). (**c**) T2-weighted, fat-suppressed image of the sciatic nerve showing fat-equivalent, hypointense fascicular lesions of the tibial and peroneal compartments in a participant with DPN. (**d**) Colour-coded FA map of the tibial and peroneal compartments of the sciatic nerve of the same participant as in (**c**). (**e**) T2-weighted, fat-suppressed image of the sciatic nerve showing both hyperintense and hypointense fascicular lesions of the tibial and peroneal compartments in a male participant with severe DPN. (**f**) Colour-coded FA map of tibial and peroneal compartments of the sciatic nerve of the same participant as in (**e**)

Using human iPSC Schwann cells, we observed that high glucose alone was able to reduce the intracellular mRNA levels of *PMP22*, *MPZ*, *PLP1* and *MBP* as well as MPZ protein expression significantly. This effect was even more pronounced with the addition of 2.5% fatty acids and could be partly ameliorated by the addition of insulin, mimicking potential conditions of the metabolic syndrome and type 2 diabetes (Fig. 2a,c,d,e,f). The decrease of intracellular mRNA levels of various myelination factors correlated significantly with the extracellular content found in the culture medium (Fig. 2a,b). In human nerve sections of the sciatic nerve of participants with and without DPN, the significant decrease of MPZ protein could be confirmed (Fig. 2g,h). Given this background, a study in humans trying to detect (extracellular) cmRNA for detecting and predicting the progression of DPN was initiated using the most promising targets derived from in vitro *MBP*, *PLP1*, *PMP22* and *MPZ*. *MBP* cmRNA was detectable in all participants, but showed a high variability. *PLP1* cmRNA could be detected in 79% of participants (96 of 122) and showed no association with DPN, whereas *PMP22* cmRNA was detectable in only 58% (71 of 122) of participants but showed a significant decrease in participants with DPN (ESM Fig. 2). *MPZ* cmRNA was detectable in all participants and relative expression levels were 7.720 ± 2.609 in healthy control participants, 6.952 ± 2.323 in participants without DPN and 4.189 ± 2.528 in participants with DPN (*p* < 0.001 vs control participants and participants w/o DPN) (Fig. 3b). Multivariate distribution analysis revealed that reduced *MPZ* cmRNA levels were significantly associated with increased NSS and NDS, and decreased NCV of *N. tibialis*, *N. peroneus* and *N. suralis* (Table 2). Additionally, various QST variables correlated significantly with decreased *MPZ* cmRNA with a focus on tests reflecting the function of the myelinated A-δ fibres and A-ß fibres (Table 2). Furthermore, MRN analysis revealed that decreased *MPZ* cmRNA was significantly associated with decreased FA, consistent with NfL (Table 2, Fig. 4).

**Table 2:** Correlation analysis of serum NfL concentrations and *MPZ* cmRNA levels with appropriate clinical/laboratory variables for all study participants

Variable	Pearson *r* of NfL	Pearson *r* of *MPZ*
Age	0.425^***^	−0.020
Sex	−0.159	−0.010
Neuropathy scores		
NDS	0.278^**^	−0.335^***^
NSS	0.124	−0.363^***^
Neurophysiology		
FA	−0.294^*^	0.589^**^
*N. tibialis* NCV	−0.249^**^	0.219^*^
*N. tibialis* amplitude	−0.248^**^	0.205^*^
*N. peroneus* NCV	−0.344^***^	0.196^*^
*N. peroneus* amplitude	−0.268^**^	0.255^**^
*N. suralis* NCV	−0.037	0.227^*^
QST		
Warm detection threshold^a^	0.203^*^	−0.234^*^
Heat pain threshold^a^	0.374^***^	−0.087
Cold pain threshold^a^	0.236^*^	−0.100
Cold detection threshold^b^	−0.256^**^	0.194^*^
Mechanical pain threshold^b^	−0.140	0.264^*^
Vibration detection threshold^c^	−0.022	0.301^**^
Mechanical detection threshold^c^	0.031	0.179^*^

Displayed are only variables that showed, for at least one biomarker, a significant association

Superscript letters indicate the mediating fibres of the extensive QST panel

^a^Small unmyelinated C fibres

^b^Thin myelinated A-δ fibres

^c^Thick myelinated A-ß fibres

*p < 0.05

**p < 0.01

***p < 0.001

### NfL

In order to study whether serum NfL is a useful marker for detecting and monitoring the progression of DPN, and also in order to test its performance against *MPZ* cmRNA, it was quantified in 119 participants. The values ranged from 3.67 to 55.2 pg/ml, with a mean value of 9.88 ± 3.72 pg/ml for healthy control participants, 12.51 ± 7.87 pg/ml in participants with type 2 diabetes without DPN and 14.69 ± 8.50 pg/ml in participants with DPN (*p* < 0.05 vs healthy control participants) (Fig. 3a). Multivariate regression analysis revealed that increased serum NfL levels were associated with increased NDS and decreased FA, as measured by MRN, and decreased NCVs of *N. tibialis*, *N. peroneus* and *N. suralis* (Table 2). Furthermore, various QST variables correlated significantly with increased serum NfL with a focus on sensory tests mediated by small unmyelinated C fibres (Table 2). MRN analysis revealed that increased serum levels of NfL were associated with decreased FA, which was reflected by either hypo- or hyper-intensive lesions of tibial and peroneal compartments of the sciatic nerve (Table 2, Fig. 4).

### Diagnostic performance

In order to test the discrimination between participants with and without DPN, receiver-operating characteristic (ROC) curve analysis was used. Using a cut-off value of 5.08, *MPZ* cmRNA achieved a sensitivity of 77.6% and specificity of 83.8%, with an AUC of 0.785, which was the best single marker regarding diagnostic performance (Table 3, Fig. 3c). In order to evaluate the improvement of already existing markers, ROC curves of combined markers were used: such as a standard model (diabetes duration, age, BMI, HbA_1c_), standard model + NCV of *N. suralis* or standard model + NCV of *N. suralis* + *MPZ* cmRNA. Using *MPZ* cmRNA, in addition to already established markers for the diagnosis of DPN, improved the diagnostic performance significantly from an AUC of 0.681 to 0.836 (Table 3, Fig. 3d). Due to the lack of discrimination between participants with DPN and without DPN, serum NfL showed only poor performance in a ROC analysis with an AUC of 0.564 (cut-off at 12.6 pg/ml).

**Table 3:** Characteristics of the ROC curve analysis for different markers (individually or combined)

Variable	AUC	95% CI	Significance (asymptotic)
*MPZ* cmRNA	0.785	0.688, 0.931	0.0001
*N. suralis* NCV	0.669	0.521, 0.818	0.036
*N. peroneus* NCV	0.597	0.458, 0.694	0.029
Diabetes duration	0.560	0.401, 0.719	0.459
HbA_1c_	0.530	0.371, 0.689	0.714
BMI	0.497	0.338, 0.656	0.971
Albuminuria	0.470	0.306, 0635	0.714
Age	0.376	0.224, 0.529	0.126
Standard model (diabetes duration, age, BMI, HbA_1c_)	0.681	0.509, 0.852	0.057
Standard model + NCV *N. suralis*	0.756	0.601, 0.910	0.007
Standard model + NCV *N. suralis* + *MPZ* cmRNA	0.836	0.711, 0.961	0.0001

### Follow-up QST

**Fig. 5:**
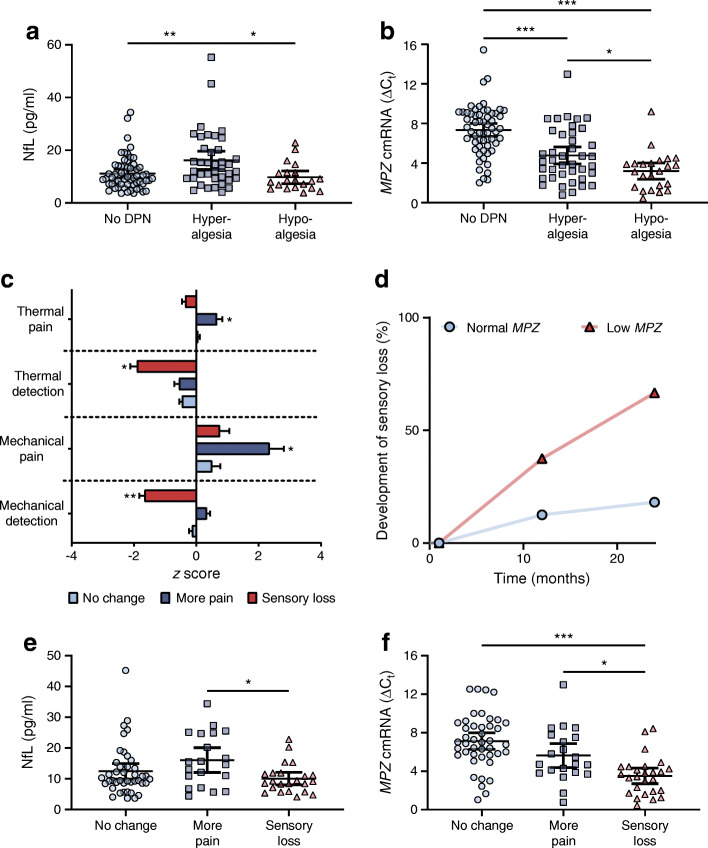
NfL and cmRNA of *MPZ* predict the hyper- and hypoalgesic phenotype in DPN. (**a**) Initial sensory profiles, based upon extensive QST, and corresponding serum NfL levels in participants at study enrolment; no DPN (*n* = 59), hyperalgesia (gain of function) (*n* = 39) and hypoalgesia (loss of function) (*n* = 20). (**b**) Initial sensory profiles, based upon extensive QST, and corresponding *MPZ* cmRNA levels in participants at study enrolment; no DPN (*n* = 59), hyperalgesia (gain of function) (*n* = 40) and hypoalgesia (loss of function) (*n* = 23). (**c**) Change in sensory profiles, based upon extensive QST, in participants 24 months after enrolment (follow-up study); ‘no change’ (*n* = 43), ‘more pain’ (*n* = 21) and ‘sensory loss’ (*n* = 26). (**d**) Kaplan–Meier curve displaying the estimated probability (%) to develop peripheral hypoalgesia (sensory loss) in two cohorts defined by *MPZ* cmRNA levels below cut-off (<5.08 ≙ low *MPZ*) and above cut-off (>5.08 ≙ normal MPZ); HR 6.519 (95% CI 2.53, 16.77); X^2^ = 13.03. (**e**) Development of hyper- (‘more pain’) or hypoalgesia (‘sensory loss’) and corresponding serum NfL levels in participants 24 months after study enrolment (follow-up) based upon extensive QST. (**f**) Development of hyper- (‘more pain’) or hypoalgesia (‘sensory loss’) and corresponding *MPZ* cmRNA levels in participants 24 months after study enrolment (follow-up) based upon extensive QST. All *MPZ* cmRNA data represent the ΔC_t_ values normalised to the geometric mean of four individual reference genes (*UBC*, *eEF1a1*, *GAPDH*, *18S*). Serum NfL levels and *MPZ* cmRNA data are displayed as mean value with 95% CI; *p < 0.05, **p < 0.01, ***p < 0.001

Baseline laboratory and clinical profiles of participants of the follow-up study were comparable to those of the baseline cohort (ESM Table 3). At enrolment participants with high serum NfL levels displayed a hyperalgesic phenotype (gain of function), whereas participants with a loss of sensorimotor function (comparable to hypoalgesia) had significantly lower *MPZ* cmRNA levels (Fig. 5a,b). After 24 months, changes in QST were assessed (*n* = 90). Forty-three participants showed no change in symptoms, 21 participants experienced ‘more pain’ and 26 participants suffered a ‘sensory loss’ determined by extensive QST assessment, which are defined as changes in thermal/mechanical pain and/or detection, respectively (Fig. 5c). Prospective evaluation revealed that high NfL protein concentrations were associated with the development of increased pain after 24 months (Fig. 5e). Participants who developed a sensory loss after 24 months had significantly lower *MPZ* cmRNA levels at enrolment as compared with the ‘no change’ and ‘more pain’ groups (Fig. 5f). In a Kaplan–Meier curve, low *MPZ* cmRNA (cut-off <5.08) was significantly associated with the outcome of ‘sensory loss’ after 24 months (HR 6.519; χ^2^ = 13.03; 95% CI 2.53, 16.77) (Fig. 5d). Multivariate regression revealed that *MPZ* expression was independently associated with the development of sensory loss when tested vs age, male sex, BMI, diabetes duration, HbA_1c_ and GFR. Participants with *MPZ* levels above the cut-off (>5.08) showed significantly lower risk (OR 0.651 [95%CI 0.39, 0.966], *p* < 0.05) for the loss of sensorimotor function (Table 4).

**Table 4:** Multivariate regression analysis of participants with DPN in the follow-up study 24 months after enrolment

Variable	More pain			Sensory loss		
	OR	95% CI	*p*	OR	95% CI	*p*
Age (years)	1.032	0.864, 1.231	NS	0.912	0.814, 1.11	NS
Male sex	5.334	0.228, 223.68	NS	0.221	0.022, 1.915	NS
BMI (kg/m^2^)	0.893	0.677, 1.311	NS	0.789	0.687, 1.11	NS
Diabetes duration (years)	1.063	0.941, 1.276	NS	0.912	0.81, 1.13	NS
HbA_1c_ (mmol/mol)	0.209	0.017, 2.844	NS	2.02	0.546, 5.889	NS
eGFR (ml min^−1^ 1.73 m^−2^)	1.001	0.894, 1.146	NS	1.01	0.925, 1.134	NS
*MPZ* cmRNA	1.184	0.589, 2.389	NS	0.651	0.398, 0.966	<0.05

### External validation

In order to validate the results of this pilot study, *MPZ* cmRNA levels were quantified in 33 external blinded serum samples of participants with type 2 diabetes of the prospective German Diabetes Study (ClinicalTrials.gov registration no. NCT01055093). Demographic, laboratory and clinical profiles of all participants showed no significant differences regarding age, sex, diabetes duration, BMI or HbA_1c_, but participants with diagnosed DPN had higher NSS and NDS as compared with those without DPN (ESM Tables 4, 5). The mean serum concentration of *MPZ* cmRNA was significantly lower in recently diagnosed participants with DPN compared with those without DPN (4.9 ± 2.9 vs 23.8 ± 7.6) (ESM Fig. 1d).

## Discussion

This study identified *MPZ* as a significantly decreased transcript in neuronal Schwann cells due to a hyperglycaemic environment which could be confirmed in human nerve sections of the sciatic nerve. In serum of participants with diabetic neuropathy, *MPZ* cmRNA was then tested against the recently established neural damage marker NfL protein. Both markers demonstrated diagnostic value, but in particular *MPZ* cmRNA displayed in a ROC curve analysis a sensitivity of 77.6% and specificity of 83.8% alone in detecting participants with DPN. Combined with a standard risk model, including diabetes duration, age, BMI, HbA_1c_ and NCV of *N. suralis*, the diagnostic performance was improved with the addition of *MPZ* cmRNA. Compared with other biomarkers for the diagnosis of DPN, *MPZ* cmRNA achieved good results [10, 11]. Neuron-specific enolase in comparison, which has been identified recently as one of the most promising candidates for detecting DPN, has been reported to reflect a sensitivity of 66.3% and a specificity of 72.5% [11, 27].

When using MRN, our study demonstrated that FA, a variable to quantify nerve fibre integrity, was associated with low levels of *MPZ* cmRNA. This may suggest that a decrease in *MPZ* cmRNA reflects the process of demyelination in A-δ/β fibres in humans, a process which has been previously shown in human imaging studies and in animal models of DPN [28–30]. This hypothesis is supported by QST variables which are mediated by myelinated A-δ and A-β fibres, and were linked to decreased *MPZ* cmRNA levels [31]. In contrast, NfL correlated mainly with QST variables mediated by small unmyelinated C fibres, which suggests a role of NfL in the maintenance of axonal function. Demyelinating neuropathy shows characteristically a reduction in NCV, whereas axonal neuropathy shows a reduction in amplitude [32]. In this study, NfL and *MPZ* were both associated with decreased NCV as well as amplitude of all major nerves of the foot, resulting from a potential combination of axonal degeneration and segmental demyelination in our cohort.

Most importantly within this context, both markers had predictive capacities. Increased NfL was able to identify participants at risk for detecting a hyperalgesic phenotype (‘gain of function’), whereas decreased *MPZ* was detected with hypoalgesia (‘loss of sensorimotor function’). Within this context, *MPZ* was more powerful as a prognostic marker in order to predict hypoalgesia, as reflected by a deficit in thermal and mechanical detection 24 months in advance. *MPZ* could therefore add a valuable advantage for the early diagnosis of nerve fibre loss in patients suffering from DPN before QST variables or NCV measurements are significantly changed.

However, the capacity of cmRNAs as blood biomarkers for diabetic complications remains elusive, because their origin, stability and practicality in clinical routine are still uncertain. In a study in individuals with diabetic retinopathy, whole blood mRNA levels of rhodopsin have been shown to reflect a benefit in differentiating the stages of the disease [14]. Recently, another study showed that the mRNA levels of *NfL* (also known as *NEFL*) in the blood of individuals with impaired glucose tolerance displayed a positive correlation with the severity of DPN [33].

This study tried to address balanced cohorts, but male diabetic individuals with DPN are overrepresented. Although there is no association of sex with NfL or MPZ, it still could be a confounder. One technical limitation of our study is the challenge to normalise *MPZ* cmRNA for each individual participant, because it is not possible to use Schwann cell-specific reference genes. This study tries to address this issue by using the mean of four individual reference genes, but artificial normalisation errors cannot be fully excluded. This is a consequence of using reference genes such as *GAPDH*, *UBC*, *eEF1a1* and *18S*, which can essentially originate from every cell within the body. When comparing *MPZ* cmRNA in participants without DPN in our initial study and the external cohort we found high variability in values. In further (multicentre) studies it is of high importance to follow an exact protocol regarding collection and processing of samples in order to avoid differences due to handling variances. Another limitation of this study is the assessment of neuropathy, which is based solely upon NSS and NDS, scoring systems which have weaknesses due to their subjectivity. The prognostic and predictive capacities of the herein described biomarkers were assessed in individuals with overt symptoms. It remains elusive as to whether the sensitivity of *MPZ* or NfL could be used in individuals with less overt symptoms.

In conclusion, this pilot study in humans found that NfL protein and *MPZ* cmRNA are non-invasive biomarkers which could act as supportive tools to diagnose DPN and potentially predict its development. Although further multicentre studies with larger patient cohorts are necessary to confirm our findings, we showed herein that axonal damage, associated with increased NfL protein in the blood, is linked to a hyperalgesic phenotype. The process of (subacute) demyelination may be characterised by decreased *MPZ* cmRNA and this could be relevant for diagnosing a hypoalgesic phenotype. Our findings support the concept that the biochemical basis of pain is different from the events involved in the loss of sensorimotor function, as has been hypothesised previously [7, 34]. Therefore, the evaluation of *MPZ* and NfL could display a significant advantage in the study of structural changes occurring in DPN and might even introduce upcoming therapeutic targets.

## Supplementary Information

ESM (PDF 509 kb)

## Data Availability

Data will be provided by the corresponding author upon reasonable request.

## Abbreviations

cmRNA: Circulating mRNA
DPN: Diabetic peripheral neuropathy
FA: Fractional anisotropy
iPSC Schwann: Human inducible pluripotent stem cells-derived Schwann
Lp(a): Lipoprotein a,
MBP: Myelin basic protein
MPZ: Myelin protein zero
MRN: Magnetic resonance neurography
NCV: Neuronal conduction velocity
NDS: Neuropathy deficit score
NfL: Neurofilament light chain
NSS: Neuropathy symptom score
PLP1: Proteolipid protein 1
PMP22: Peripheral myelin protein 22
qPCR: Quantitative PCR
QST: Quantitative sensory testing
ROC: Receiver-operating characteristic
