# Supplementary material for: Neuron-specific biomarkers predict hypo- and hyperalgesia in individuals with diabetic peripheral neuropathy
Source: Diabetologia. 2021 Sep 3;64(12):2843–55. doi: 10.1007/s00125-021-05557-6 (PMC8563617; doi:10.1007/s00125-021-05557-6)
Supplement: Supplementary file 1 — (PDF 509 kb) [file 125_2021_5557_MOESM1_ESM.pdf]

Electronic Supplementary Material (ESM)

ESM Methods

Assessment of DPN

For NDS, a score was derived from examination of the ankle reflex, vibration, pin-prick and temperature (cold tuning fork) sensation at the great toe and categorized as follows: no deficit (0-2 points); mild (3-5 points); moderate (6-8 points), and severe deficit (9-10 points) (1). For NSS, a score of two was assigned if patients experienced a pain or discomfort in the legs such as burning, numbness or tingling, whereas fatigue, cramping or aching was scored with one. The presence of those symptoms in the feet was assigned a score of two, the calves a score of one, and elsewhere no score. A score of one was added if the symptoms had ever woken the patient from sleep. Furthermore, the patients were asked if any manoeuvre could reduce the symptoms: walking was assigned a score of two, standing was a score of one and sitting or lying down a score of zero (1). All scores were added and categorized as follows: no symptoms (0-2 points); mild (3-4 points); moderate (5-6 points) and severe symptoms (7-10 points).

For QST, a protocol was established using seven different tests with 13 different categories for determination of gain of function (hyperalgesia) or sensory loss (neuropathic deficits) as described previously (2). Included categories were: cold detection threshold (CDT); warm detection threshold (WDT), thermal sensory limen (TSL); cold pain threshold (CPT); heat pain threshold (HPT); pressure pain threshold (PPT); mechanical pain threshold (MPT); mechanical pain sensibility (MPS); wind-up ratio (WUR); mechanical detection threshold (MDT); vibration detection threshold (VDT); dynamic allodynia (DMA), and paradox heat sensation (PHS). Small unmyelinated C-fibers mediate temperature detection and pain (WDT, HPT, CPT, PPT), thin myelinated A-δ-fibers mediate cold detection and pinprick pain (CDT, MPT, MPS), while thick myelinated (large) A-β-fibers mediate mechanical and vibration thresholds (MDT, VDT). TSL, PHS, DMA and WUR are mediated in combination of different fibers.

All QST parameters, (except DMA and PHS) were adjusted for age and gender by calculating Z-scores based on standard values by over 180 healthy people. In order to evaluate the association between MPZ cmRNA, serum NfL levels and QST parameters, different sensory profiles were defined: "mechanical detection" (increased/decreased MDT, VDT); "mechanical pain" (increased/decreased MPS, MPT, PPT); "thermal detection" (increase/decrease WDT, CDT, TSL); “thermal pain” (increased/decreased CPT, HPT), and “mechanical hyperalgesia” (gain of MPT, MPS), which were both combined as "pain" group.

The electrophysiological examination was performed in 119 patients using Viking IV electromyography system (Viasys Healthcare GmbH) on peroneal, tibial, and sureal nerves. Skin temperature was minimum 32 °C during examination. For all nerves, amplitude [µV], latency [ms], and nerve conduction velocity [m/sec] were measured. All tests were performed on one foot to detect distal neuropathic deficit and one hand as intra-patient control area. All investigators performing QST were trained and certified by the Department of Neurophysiology at the University Hospital of Mannheim. DPN was determined by a score of three or higher in the NDS and NSS and abnormal electrophysiological examinations in two different nerves. All tests were performed at baseline and repeated after 12 and 24 months.

In addition, 37 patients screened for DPN underwent diffusion-weighted high-resolution MRN of the right thigh in a 3.0 Tesla MRI scanner (Magnetom TIM-TRIO; Siemens Healthcare; 15-channel transmit-receive extremity coil) at baseline in order to determine the sciatic nerve´s fractional anisotropy (FA), a dimensionless quantity that measures directed diffusion, with values between 0 (isotropic diffusion) and 1 (diffusion in only one direction). In previous studies, the sciatic nerve´s FA has been proven to be a sensitive indicator for the nerve’s structural integrity as compared to radial and axial diffusivity (3, 4, 5). MRN analysis was based upon the free will of the individual participant as well as the availability of the MRN system. The following sequence protocol was applied: 1) Axial high resolution T2-weighted turbo spin echo (TSE) 2D sequence with spectral fat saturation, and the following parameters: relaxation time (TR) = 5970 ms, echo time

(TE) = 55ms, field of view (FOV) = 160 x 160 mm^2, matrix size = 512 x 512, slice thickness = 4 mm, interslice gap = 0.8 mm, voxel size = 0.3 x 0.3 x 4.0 mm^3, 3 averages, 24 slices, 24 acquired images; 2) Axial fat-suppressed, diffusion-weighted 2-dimensional echo-planar sequence with the following parameters: TR = 5100 ms; TE=92.8 ms; b = 0 and 1000 s/mm^2; directions = 20; field of view 160 x 160 mm^2; matrix size 128 x 128; slice thickness 4 mm; voxel size 1.3 x 1.3 x 4 mm^3; no interslice gap, 3 averages, 24 slices, 1512 acquired images. Both sequences were centered on the sciatic nerve's bifurcation in all participants. All images were pseudonymized immediately after acquisition. After automated co-registration of both sequences, the nerves' FA was calculated in an automated approach using a Food and Drug Administration (FDA) approved processing software provided by Nordic Neurolab (Bergen, Norway) programmed for automated reconstruction of fibre tracts in diffusion weighted imaging (6). A total number of 37 x 1536 = 56832 images were analyzed accordingly. A graphic overview of the process of image co-registration and nerve segmentation is given in Figure 4.

Direct RT-qPCR from Serum

The direct extraction of cmRNA from serum samples and the RT-qPCR has been described partly previously (7-9). In detail, an amount of 20 µl serum was mixed with 20 µl of a preparation buffer containing 5% Tween®20 (polysorbate), 2 mmol/l EDTA, 25 mmol/l Tris-HCl. The mixture was directly converted into cDNA using a high capacity cDNA reverse transcription (RT) kit including 10x RT Buffer, 25x dNTP Mix (100 mmol/l), 10x RT Random Primers, MultiScripe® reverse transcriptase and RNase inhibitor (all from Thermo Fisher Scientific). The resulted cDNA (yield between 1 - 2.5 ng/µl) was used undiluted for qPCR, which was performed in duplicates for each gene per patient using QuantiNova SYBR® Green (QIAGEN) and a LightCycler® 480 instrument II (Roche Applied Science). Signals of amplified products were verified using melting curve analysis, and mRNA levels were normalised to the geometric mean of four individual reference genes (Ubiquitin C - UBC; 18S Ribosomal RNA - 18S; Glyceraldehyde-3-phosphate dehydrogenase - GAPDH; Eukaryotic translation

elongation factor 1 alpha 1 - eEF1a1). Circulating mRNA (cmRNA) levels for four myelin-specific genes (Peripheral Myelin Protein 22 - *PMP22*; Proteolipid 1 - *PLP1*; Myelin Basic Protein - *MBP*; Myelin Protein Zero - *MPZ*) were calculated by quantification cycler (C_t) and normalised to the four reference genes using the 2-ΔΔC_t method as described previously (8). In detail, the mean C_t value of the sample duplicates was subtracted by the geometric mean of the C_t values of all four reference genes (ΔC_t). The normalised ΔC_t value was then calculated with the formula as described below:

$$\text{Relative cmRNA level } = 2^{-\Delta Ct}$$

Example calculation for a control subject:

geometric mean reference genes quantification cycler:

$C_t = 22.9$

Mean of *MPZ* quantification cycle:

$C_t = 20.2$

$\Delta C_t = 23.8 – 26.5 = -2.7$

$$\text{cmRNA level} = 2^{-(-2.7)} = 6.5$$

Amplification efficiencies were tested by obtaining C_t values of serial dilutions of a specific target using a pooled serum sample (five control subjects). By plotting C_t values against the mRNA copy number (standard curve), we used a linear regression and the equation $E = -1 + 10^{(-\frac{1}{slope})}$ to calculate the PCR efficiency (ESM Fig. 1).

External validation study

Thirty-three volunteers with recently diagnosed type 2 diabetes were participants of the prospective German Diabetes Study (GDS) (ClinicalTrials.gov Identifier: NCT01055093). The cohort profile, study design, ethics approval, and the assessment of DPN were described previously (11, 12). The data were assessed using the nonparametric Mann-Whitney U test and Spearman rank correlation. Logistic regression analyses were performed to determine an association between DPN and *MPZ* cmRNA concentration, after adjustment for sex, age, BMI, current smokers, and diabetes duration. Multiple linear regression analyses with adjustments for sex, age, BMI, current smokers, and diabetes duration were performed to determine an association between neurophysiological parameters and *MPZ* cmRNA concentration. Analyses were performed using SPSS version 22.0 software.

Quantification of Neurofilament Light Chain protein

For analysis of NfL in serum samples a highly sensitive Simoa immunoassay (Quanterix, Billerica, MA, USA) was used. The basic assay principle of this type of assay is very similar to ELISAs or Luminex sandwich immunoassays. Briefly, capture antibodies are immobilized on paramagnetic microspheres, incubated with sample and biotinylated detection antibodies. In a final incubation step the microspheres are incubated with β-galactosidase-labeled streptavidin for introduction of an enzymatic label to the immunocomplex. Read-out is performed in Simoa discs carrying microarrays with femtoliter-sized wells, where a single immunocomplex is sufficient to generate a fluorescent signal by enzymatic substrate conversion to be detected by the Simoa analyzer. This single molecule read-out is leading to the increased sensitivity compared to conventional immunoassays (13). NfL was measured from 4-fold diluted serum samples using the Simoa NF-LIGHT kit (Quanterix). Reagents and calibrators were provided ready-to-use. Sample processing and analysis was done using a HD-X

analyzer (operated under Simoa software version 1.6.1905.300, both from Quanterix). The Simoa software was also used for back-calculation of analyte concentrations from measurement data.

Statistical analysis

Statistical data analysis was performed using GraphPad Prism 7 (GraphPad Software Inc.) and SPSS Version 23.0 (IBM) and can be found in detail in the electronic supplementary material. For normally distributed parameters, one-way ANOVA analysis was used for inter-group comparison. For non-gaussian distribution, Mann-Whitney test was used for comparison of two groups and Wilcoxon test and/or Kruskal-Wallis test for more than two groups. Correlations between circulating mRNA and parameters of incident DPN were estimated using Bonferroni-corrected Pearson correlation coefficients with corresponding P values. A multivariate logistic regression analysis was performed for- and backward to analyze associations between clinical parameters and circulating mRNA levels. QST results were stratified for test site, age and gender as described previously (14) and the resulting z-scores were used for statistical analysis. Logistic regression model was used for incident DPN and circulating mRNA and predicted probability values were used for ROC curve analysis. The Youden index (sensitivity+specificity-1) was used for the calculation of a cut-off value with the best performance comparable to the method of simultaneous maximization of sensitivity and specificity. For ROC-curve analyses of combined markers a binary logistic regression was performed, whereas single covariates (age, BMI, HbA_{1c}, *MPZ* cmRNA etc.) were merged in order to obtain probabilities for a DPN outcome with combined variables.

The intended sample size for each group was calculated with a two-tailed t-test with an estimated power of 0.8 (for both groups) and sigma values of 0.8. For calculating the sample size the following formula was used and it was on the supposition that "control group" consisted of healthy controls and T2D without DPN:

$$Sample\ Size = \frac{r+1}{r} \frac{SD^2 (Z_{ß} + Z_{\frac{a}{2}})^2}{d^2}$$

r = control to cases ratio

SD = standard deviation (determined in small pilot study ≙ 3.79)

Zß = standard normal variate for power (80% power ≙ 0.84)

Za/2 = standard normal variate (for $p<0.05$ type 1 error ≙ 1.96)

d = expected mean difference between case and control (determined in small pilot study ≙ 2.9)

$$Sample\ Size = \frac{2}{1}\frac{3.79^2(0.84+1.96)^2}{2.9^2} = 26.8$$

References

1. M. J. Young, A. J. M. Boulton, A. F. Macleod, D. R. R. Williams, P. H. Sonksen, A multicentre study of the prevalence of diabetic peripheral neuropathy in the United Kingdom hospital clinic population, Diabetologia 36, 150–154 (1993).
2. R. Rolke, R. Baron, C. Maier, T. R. Tölle, et al. Quantitative sensory testing in the German Research Network on Neuropathic Pain (DFNS): Standardized protocol and reference values:, Pain 123, 231–243 (2006).
3. Vaeggemose M, Haakma W, Pham M, et al. (2020) Diffusion tensor imaging MR Neurography detects polyneuropathy in type 2 diabetes. *J Diabetes Complications* 34: 107439
4. Jende JME, Kender Z, Mooshage C, et al. (2021) Diffusion Tensor Imaging of the Sciatic Nerve as a Surrogate Marker for Nerve Functionality of the Upper and Lower Limb in Patients With Diabetes and Prediabetes. *Front Neurosci* 15: 642589
5. M. Vaeggemose, M. Pham, S. Ringgaard, H. Tankisi, N. Ejskjaer, S. Heiland, P. L. Poulsen, H. Andersen, Magnetic Resonance Neurography Visualizes Abnormalities in Sciatic and Tibial Nerves in Patients With Type 1 Diabetes and Neuropathy, Diabetes 66, 1779–1788 (2017).
6. L. Orsingher, S. Piccinini, G. Crisi, Differences in dynamic susceptibility contrast MR perfusion maps generated by different methods implemented in commercial software, J Comput Assist Tomogr 38, 647–654 (2014).
7. Zhang X, Yang X, Zhang Y, et al. (2015) Direct Serum Assay for Cell-Free Bmi-1 mRNA and Its Potential Diagnostic and Prognostic Value for Colorectal Cancer. *Clinical Cancer Research* 21: 1225–1233
8. Asaga S, Kuo C, Nguyen T, Terpenning M, Giuliano AE & Hoon DSB (2011) Direct Serum Assay for MicroRNA-21 Concentrations in Early and Advanced Breast Cancer. *Clinical Chemistry* 57: 84–91
9. Zhang X, Wang C, Wang L, et al. (2012) Detection of circulating Bmi-1 mRNA in plasma and its potential diagnostic and prognostic value for uterine cervical cancer. *International Journal of Cancer* 131: 165–172
10. Livak KJ & Schmittgen TD (2001) Analysis of Relative Gene Expression Data Using Real-Time Quantitative PCR and the $2^{-\Delta\Delta CT}$ Method. *Methods* 25: 402–408
11. Szendroedi J, Saxena A, Weber KS, et al. (2016) Cohort profile: the German Diabetes Study (GDS). *Cardiovasc Diabetol* 15: 59
12. Ziegler D, Papanas N, Zhivov A, et al. (2014) Early detection of nerve fiber loss by corneal confocal microscopy and skin biopsy in recently diagnosed type 2 diabetes. *Diabetes* 63: 2454–2463

13. Kopf S, Groener JB, Kender Z, et al. (2018) Deep phenotyping neuropathy: An underestimated complication in patients with pre-diabetes and type 2 diabetes associated with albuminuria. *Diabetes Research and Clinical Practice* 146: 191–201
14. Rissin DM, Kan CW, Campbell TG, et al. (2010) Single-molecule enzyme-linked immunosorbent assay detects serum proteins at subfemtomolar concentrations. *Nature Biotechnology* 28: 595–599

ESM Table 1 Primer sequences.

Target		Gen Bank ID	Primer Bank ID*	Amplicon Size [bp]	Forward (5`>3`)	Reverse (5`>3`)
Human	*GAPDH*	2597	378404907c2	101	ACAACTTTGGTATCGTGGAAGG	GCCATCACGCCACAGTTTC
	18S	6222	14165467c2	93	ATCACCATTATGCAGAATCCACG	GACCTGGCTGTATTTTCCATCC
	UBC	7316	305632811c1	117	CTGGAAGATGGTCGTACCCTG	GGTCTTGCCAGTGAGTGTCT
	eEF1a1	1915	83367078c1	89	GCTGAGCGTGAACGTGGTAT	CCTGGGGCATCAATGATAGTCA
	PLP1	5354	192449446c1	109	ACCTATGCCCTGACCGTTG	TGCTGGGGAAGGCAATAGACT
	MBP	4155	307159a2	90	TCGGCTCACAAGGGATTCAAG	TGATCCAGAGCGACTATCTCTTC
	MPZ	4359	295391070c3	130	CTCTCAGGTCACGCTGTATGT	GCAGTACCGAACCACGTAGAAA
	PMP22	5376	24430162c2	133	GATCCTGTCGATCATCTTCAGC	AGCACTCATCACGCACAGAC
	NEFL	4747	197927150c1	200	ATGAGTTCCTTCAGCTACGAGC	CTGGGCATCAACGATCCAGA
	ß-Actin	81822	-	197	ACAACCTTCTTGCAGCTCC	ACCCATACCCACCATCACAC

*

Primer Bank ID`s are provided by the PrimerBank of the Harvard Medical School, which is a public resource for human and murine PCR primers. As described on the website (https://pga.mgh.harvard.edu/primerbank/)

ESM Results

ESM Table 2 Characteristics of participants in human nerve study.

Parameter	Non-diabetic	Type-2 diabetic with DPN
Age (Years)	67 ± 5	72 ± 12
Sex (M/F)	2/2	4/1
BMI (kg/m^2)	22.9 ± 4.6	25.9 ± 1.7
Complications		
• CVD	2/4	5/5
• Retinopathy	0/4	0/5
• Nephropathy	1/4	2/5
• Neuropathy	0/4	5/5
Reason for Amputation	Ischemia/infection	Ischemia/infection

Data are mean ± SD

ESM Table 3 Baseline laboratory and clinical profiles of participants of the follow up study 24 months after enrollment.

Parameter	no change (*n* = 43)	more pain (*n* = 21)	sensory loss (*n* = 26)
Age (years)	58.9 ± 13.01	62.0 ± 10.24	61.9 ± 9.36
Sex (f/m)	20/43	10/21	11/26
Diabetes duration (years)	4.4 ± 7.4	5.7 ± 9.3	6.3 ± 6.9
BMI (kg/m^2)	28.8 ± 4.3	27.5 ± 5.1	27.8 ± 3.1
HbA_{1c} (mmol/mol)	51.9 ± 17.5	51.9 ± 9.9	53.0 ± 13.1
HbA_{1c} (%)	6.9 ± 1.6	6.9 ± 1.0	7.0 ± 1.2
eGFR (ml min^{-1} 1.73 m^{-2})	88.1 ± 14.7	92.3 ± 12.2	91.6 ± 7.2
hsCRP (mg/l)	3.0 ± 4.3	3.1 ± 3.4	1.8 ± 1.8
Triacylglycerols (mmol/l)	1.87 ± 1.15	1.40 ± 0.70	2.35 ± 2.04
Cholesterol (mmol/l)	4.65 ± 0.95	4.58 ± 0.93	5.09 ± 1.38
BP systolic (mmHg)	134.0 ± 12.9	141.9 ± 22.9	134.7 ± 11.1
BP diastolic (mmHg)	82.2 ± 8.1	81.2 ± 7.3	83.0 ± 8.6
NSS	1.9 ± 3.5	2.8 ± 3.8	4.0 ± 3.4
NDS	2.8 ± 3.0	2.0 ± 2.6	3.4 ± 2.6
Oral antidiabetics	12 (28)	9 (43)	10 (38)
Insulin therapy	11 (26)	8 (38)	7 (27)
RAAS inhibitors	11 (26)	10 (48)	11 (42)
Beta blockers	9 (21)	5 (24)	6 (23)
ASA	9 (21)	2 (10)	5 (19)
Statins	14 (33)	8 (38)	5 (19)
Diabetic nephropathy	4 (9)	2 (10)	3 (12)
Diabetic retinopathy	-	-	-

Data are mean ± SD or *n* (%)

- data is not available

ESM Table 4 Demographic, laboratory and clinical profiles of participants of the external cohort.

**Parameters**	**T2D w/o DPN (*n* = 17)**	**T2D with DPN (*n* = 16)**	***p***
Age (years)	53.4 ± 6.2	56.4 ± 7.6	0.228
Sex (f/m)	3/14	5/11	0.438
Diabetes duration (years)	17.5 ± 95	13.4 ± 81	0.198
BMI (kg/m^2)	31.0 ± 5.0	34.9 ± 7.6	0.088
Fasting glucose (mmol/l)	7.33 ± 1.56	8.06 ± 2.44	0.326
HbA_{1c} (mmol/mol)	43.7 ± 9.6	50.7 ± 12.0	0.072
HbA1c (%)	6.15 ± 0.87	6.79 ± 1.09	0.072
Triacylglycerols (mmol/l)	1.82 ± 0.86	2.08 ± 1.39	0.522
Cholesterol (mmol/l)	5.33 ± 0.78	5.41 ± 0.91	0.750
BP systolic (mmHg)	134 ± 18	139 ± 11	0.389
BP diastolic (mmHg)	76.2 ± 8.2	77.2 ± 8.3	0.728
NSS	0.00 ± 0.00	3.31 ± 2.89	>0.001
NDS	0.00 ± 0.00	4.31 ± 2.87	>0.001

Data are mean ± SD

ESM Table 5 Multiple linear regression analysis of MPZ cmRNA levels and corresponding clinical/laboratory parameters for all study participants of the external validation cohort.

**Nerve function test**	***β***	***p***
Peroneal MNCV	0.702	0.0002
Median MNCV	0.506	0.004
Ulnar MNCV	0.564	0.002
Sural SNCV	0.572	0.006
Median SNCV	0.506	0.004
Ulnar SNCV	0.564	0.002
Sural SNAP	0.727	0.0001
Ulnar SNAP	0.665	0.001
Warm TDT on foot	-0.621	0.001
Cold TDT on foot	0.384	0.042

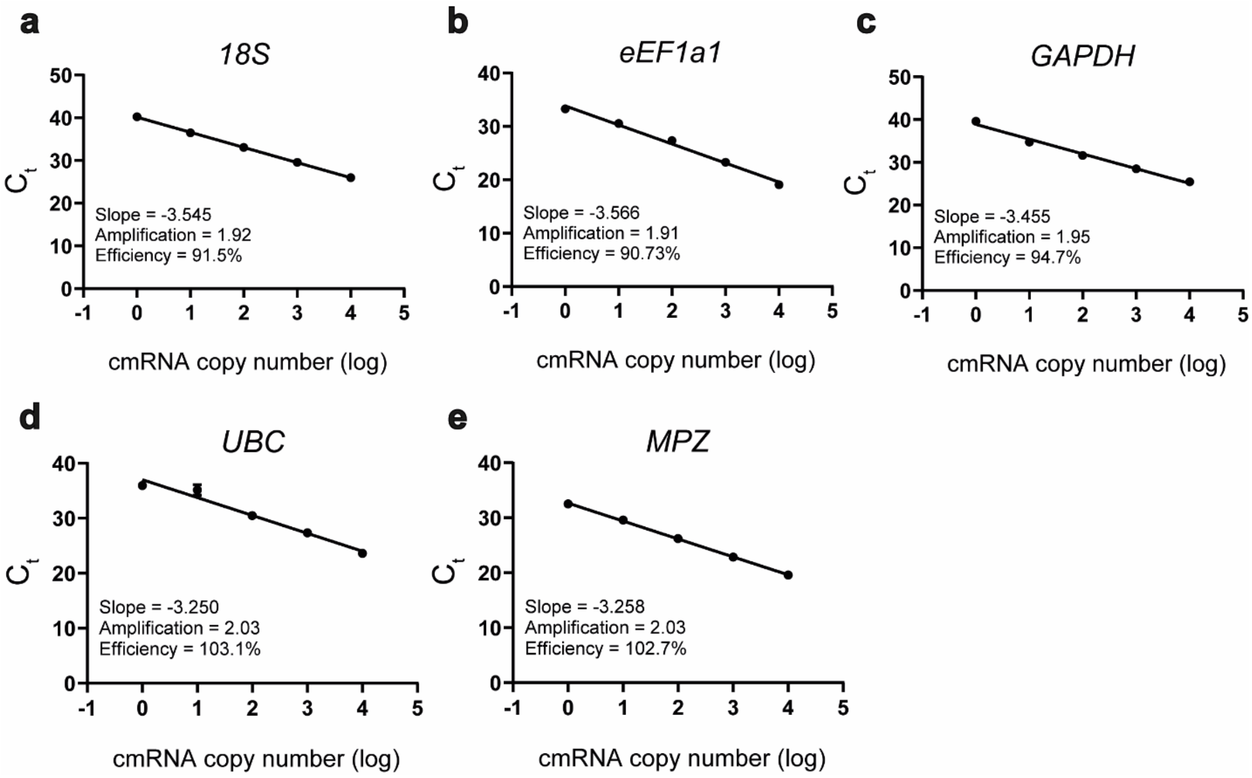

ESM Fig. 1 Amplification efficiencies of the primer pairs used for cmRNA analysis. (**a** - **e**) Standard calibration curve for *18S*, *eEF1a1*, *GAPDH*, *UBC*, and *MPZ* cmRNA in a pooled human serum sample (*n* = 5) using a linear regression model.

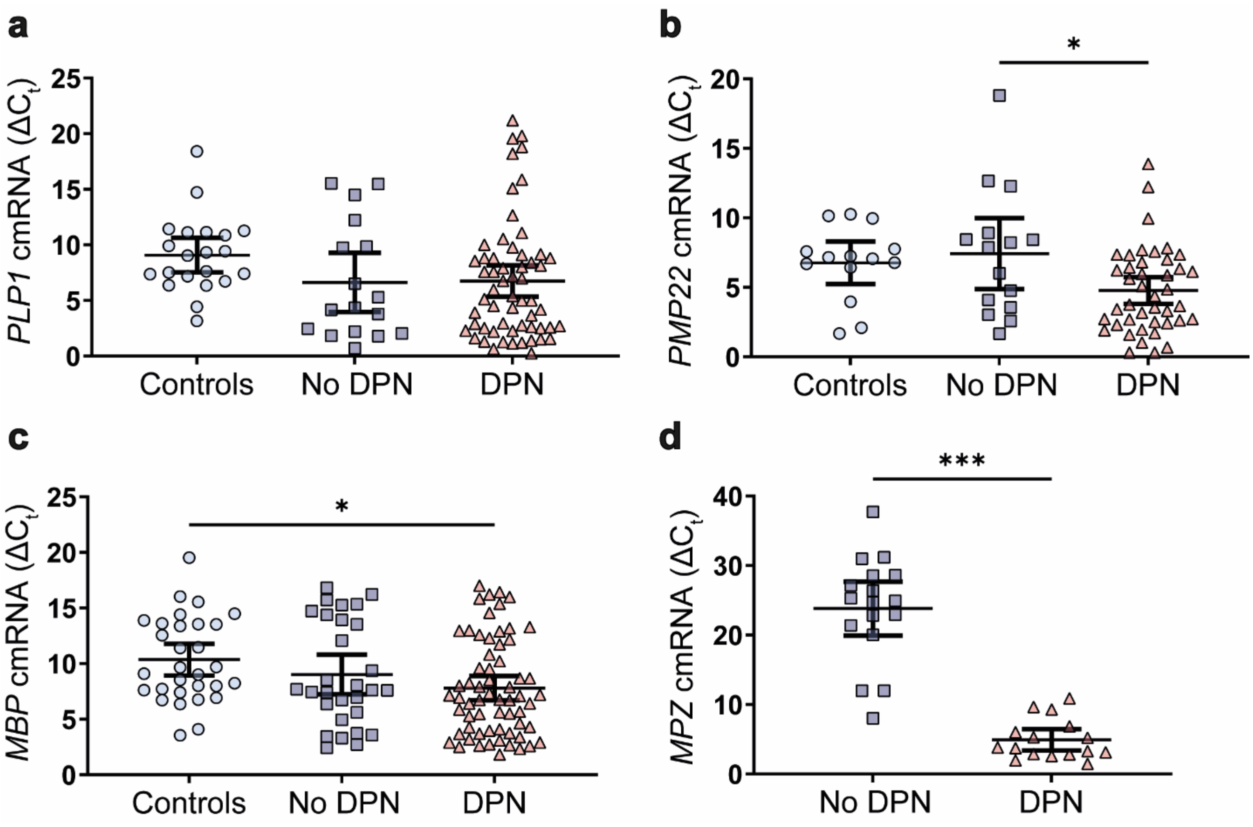

ESM Fig. 2 Quantification of circulating mRNA (cmRNA) analysis of Proteolipid 1 (*PLP1*), Peripheral Myelinating Protein 22 (*PMP22*) and Myelin Basic Protein (*MBP*). (**a**), Quantification of *PLP1* cmRNA in control patients (n = 21), patients without DPN (n = 17) and patients with diagnosed DPN (n = 58). (**b**), Quantification of *PMP22* cmRNA in control patients (n = 14), patients without DPN (n = 15) and patients with DPN (n = 42). (**c**), Quantification of *MBP* cmRNA in control patients (n = 63), patients without DPN (n = 29) and patients with DPN (n = 42). (**d**) Quantification of *MPZ* cmRNA in a blinded external cohort of patients with DPN (n = 16) and patients without DPN (n = 17). All cmRNA data represent the ΔC_t values normalised to the geometric mean of four individual reference genes (*UBC*, *eEF1a1*, *GAPDH*, *18S*) and displayed as mean value with 95% CI; * $p < 0.05$; *** $p < 0.001$.
